# Coronary artery disease, left ventricular function and cardiac biomarkers determine all-cause mortality in cancer patients—a large monocenter cohort study

**DOI:** 10.1007/s00392-022-02001-6

**Published:** 2022-03-21

**Authors:** Daniel Finke, Markus B. Heckmann, Susanna Wilhelm, Lukas Entenmann, Hauke Hund, Nina Bougatf, Hugo A. Katus, Norbert Frey, Lorenz H. Lehmann

**Affiliations:** 1 Department of Cardiology, Heidelberg University Hospital, Im Neuenheimer Feld 410, 69120 Heidelberg, Germany; 2 German Centre for Cardiovascular Research (DZHK) Partner Site, Heidelberg/Mannheim, Germany; 3 Nationales Tumorzentrum Heidelberg (NCT), Heidelberg, Germany; 4 Deutsches Krebsforschungszentrum Heidelberg (DKFZ), Heidelberg, Germany

**Keywords:** Cardio-oncology, Cancer patient risk stratification, Cardiac assessments in cancer patients, Shared risk factors, Atherosclerosis

## Abstract

Cancer patients are at risk of suffering from cardiovascular diseases (CVD). Nevertheless, the impact of cardiovascular comorbidity on all-cause mortality (ACM) in large clinical cohorts is not well investigated. In this retrospective cohort study, we collected data from 40,329 patients who were subjected to cardiac catherization from 01/2006 to 12/2017 at University Hospital Heidelberg. The study population included 3666 patients with a diagnosis of cancer prior to catherization and 3666 propensity-score matched non-cancer patients according to age, gender, diabetes and hypertension. 5-year ACM in cancer patients was higher with a reduced left ventricular function (LVEF < 50%; 68.0% vs 50.9%) or cardiac biomarker elevation (high-sensitivity cardiac troponin T (hs-cTnT; 64.6% vs 44.6%) and N-terminal brain natriuretic peptide (NT-proBNP; 62.9% vs 41.4%) compared to cancer patients without cardiac risk. Compared to non-cancer patients, NT-proBNP was found to be significantly higher (median NT-proBNP cancer: 881 ng/L, IQR [254; 3983 ng/L] vs non-cancer: 668 ng/L, IQR [179; 2704 ng/L]; *p* < 0.001, Wilcoxon-rank sum test) and turned out to predict ACM more accurately than hs-cTnT (NT-proBNP: AUC: 0.74; hs-cTnT: AUC: 0.63; *p* < 0.001, DeLong’s test) in cancer patients. Risk factors for atherosclerosis, such as diabetes and age (> 65 years) were significant predictors for increased ACM in cancer patients in a multivariate analysis (OR diabetes: 1.96 (1.39–2.75); *p* < 0.001; OR age > 65 years: 2.95 (1.68–5.4); *p* < 0.001, logistic regression). Our data support the notion, that overall outcome in cancer patients who underwent cardiac catherization depends on cardiovascular comorbidities. Therefore, particularly cancer patients may benefit from standardized cardiac care.

**Graphical abstract:**

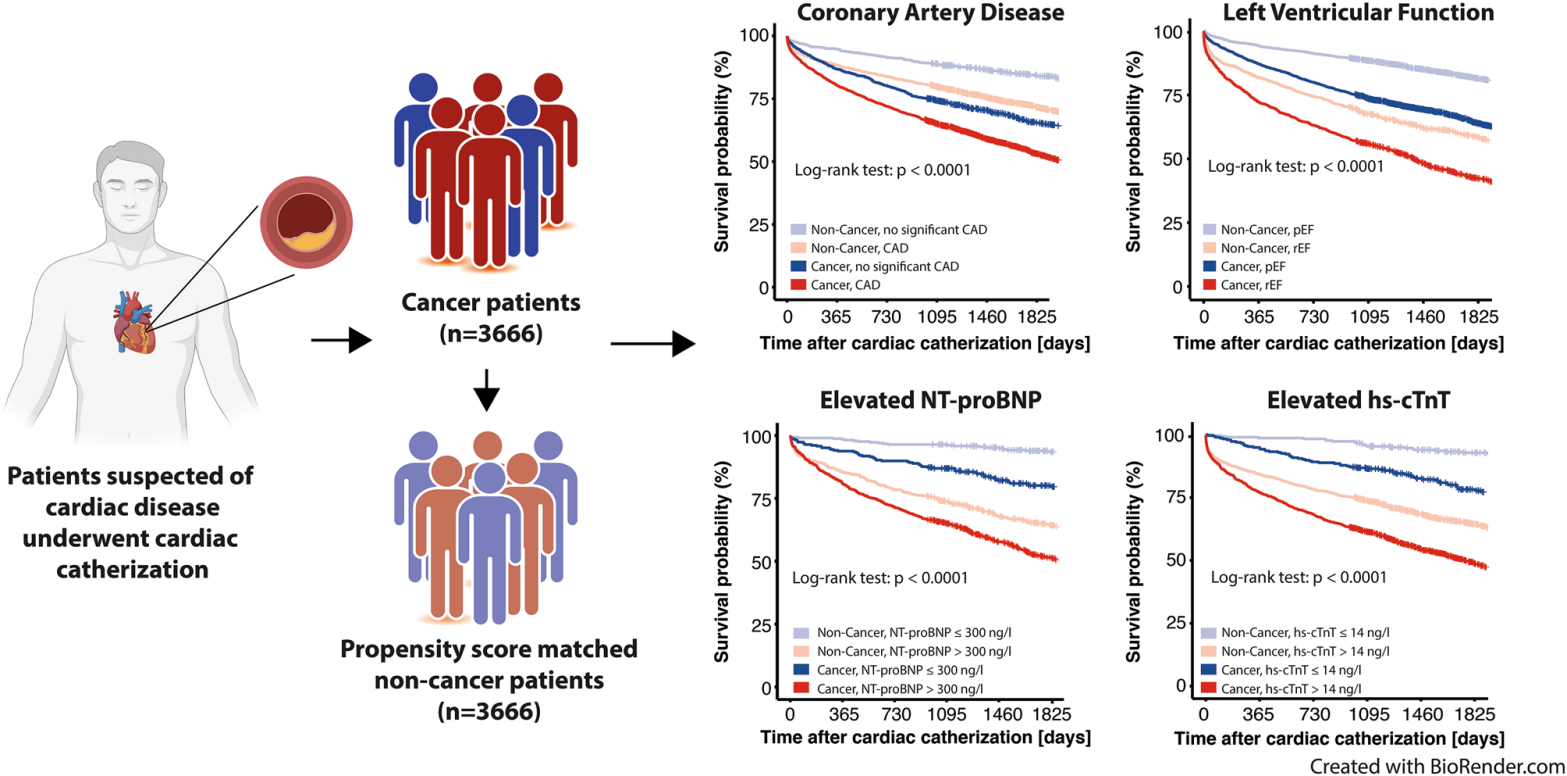

**Supplementary Information:**

The online version contains supplementary material available at 10.1007/s00392-022-02001-6.

## Introduction

Cardiovascular disease (CVD) as well as cancer are leading causes of death in the western world. The co-occurrence of both is common and defines a high-risk patient cohort with high mortality rates [1]. In community-based retrospective data, cancer patients with CVD have a higher all-cause mortality (ACM) compared to patients suffering from either oncological or cardiovascular diseases alone [2]. Specific cardiovascular complications, such as stroke or bleedings, are additionally frequent in cancer patients and are relevant for the patients’ survival [3]. As one example, a population-based analysis in the US recently finds breast, prostate and bladder cancer patients to have the highest incidences of death due to CVD [4].

Investigating the connection of cancer and CVD, the occurrence of both, cancer and cancer-related side effects are higher in patients with CVD [5]. Vice versa, CVD is found to be an important co-factor leading to neoplastic diseases [6]. Inflammatory cytokines [7], sympathetic nervous system activation [8] and inhibition of the renin–angiotensin–aldosterone system (RAAS) are other connecting mechanisms of the two diseases [9, 10].

Besides that, diabetes and arterial hypertension are common risk factors for CVD and cancer [11]. Coronary artery disease (CAD) is one of the most prevalent manifestations in response to the accumulation of CVD risk factors [12]. Due to overlapping risk factors for CVD and cancer [11], there is a high incidence of CAD in cancer patients and cancer survivors which is associated with a poor outcome [13, 14]. Apart from that, only little is known about the influence of CAD, as an independent factor, on ACM in a clinical cohort of cancer patients.

More importantly, cardiovascular risk stratification in cancer patients and cancer patients with CAD, still remains challenging. Currently, in accordance with the guidelines of the ESC [15] and ASCO [2], surveillance strategies mainly rely on non-invasive imaging, such as echocardiography and cardiac MRI [16].

The primary objective in the present study is to determine the impact of coronary artery disease, cardiac biomarkers and left ventricular function on ACM in cancer patients that underwent cardiac catherization. Further, we aim to evaluate differences in the occurrence of CAD, reduced left ventricular ejection fraction (LVEF) as well as levels of cardiac biomarkers of cancer and matched non-cancer patients. We hypothesize that cardiovascular complications in cancer patients are possibly caused by ischemia-independent mechanisms due to the systemic disease and its treatment.

Among all cancer patients, we found elevated levels of N-terminal brain natriuretic peptide (NT-proBNP) in comparison to matched non-cancer patients. Biomarker elevation (NT-proBNP or high-sensitivity cardiac troponin T (hs-cTnT)) as well as a reduced LVEF were associated with a poor prognosis. In breast cancer patients, CAD had an additional prognostic value.

## Materials and methods

### Study design and patients

This cohort study was conducted retrospectively at University Hospital Heidelberg. Data were acquired from 40,329 patients who underwent cardiac catherization at University Hospital Heidelberg between January 1, 2006 and December 31, 2017.

After identifying cancer patients with an initial diagnosis before cardiac catherization, non-cancer patients were matched according to age, gender, diabetes and arterial hypertension as a control group. Cancer patients were further subgrouped according to their cancer entity. We focused on the five most prevalent entities in our cohort for further analysis.

The study protocol was approved by the ethics committee of the Medical Faculty of the University of Heidelberg (S-286/2017, 390/2011). The investigation conformed with the principles outlined in the *Declaration of Helsinki.*

### Data acquisition

Patient specific data were extracted from electronic medical records including demographic data, laboratory results (including creatinine, hemoglobin (Hb), C-reactive protein (CRP), leucocyte count, hs-cTnT and NT-proBNP) and angiographic measurements using the cardiac Research Data Warehouse (RWH). LVEF was assessed during cardiac catherization. The highest laboratory values that were assessed between 7 days prior to 7 days after the cardiac catherization were considered for further analysis. The oncological diagnosis (ICD-10 code), date of diagnosis and treatment were retrieved from the Clinical Registry of the National Centre for Tumor Diseases (NCT) Heidelberg. Data of all-cause mortality and date of death were obtained from the registration office from the patients’ place of residence. Follow-up of ACM was analyzed by October 1, 2020.

Measurement of hs-cTnT in plasma samples was performed using the Elecsys® Troponin T high sensitive hs-cTnT assay (Roche Diagnostics) in the central laboratory at Heidelberg University Hospital. Limit of blank (LoB), Limit of detection (LoD), 10% coefficient of variation (CV) and 99th percentile cut-off values for the hs-cTnT assay were 3, 5, 13 and 14 ng/L. NT-proBNP was measured at the same timepoint using the Stratus® CS Acute Care™ NT‐proBNP assay (Siemens AG, Berlin and Munich, Germany).

### Definitions

Impairments in the LVEF were defined as previously published and recommended in the guidelines of the European Society of Cardiology (ESC) from 2021. [17] Coronary stenosis ≥ 50% was used as cut-off for the occurrence of CAD. [18] Elevated hs-cTnT levels were determined as levels above the 99th percentile (14 ng/L) [19] and elevated NT-proBNP as levels above the rule-out criterion of heart failure (300 ng/L) [20].

### Statistical analysis

To compare continuous variables, we used the Wilcoxon-rank sum test. The variables were presented as median values with interquartile range (IQR). Dichotomic data were compared using the chi-squared test and is presented as numbers of events and percentages. A confidence interval of 95% was considered significant.

1:1 propensity score matching was performed with the use of *MatchIt* (version 4.1.0) according to age, gender, diabetes and arterial hypertension. Nearest neighbor matching was performed. The distance measure was estimated via logistic regression. No units were discarded before matching. Thus, we were able to obtain an adequate balance of propensity scores between the cancer and non-cancer group (Supplemental Fig. 1).

Logistic regression analyses were performed in R, version 4.0.2., using *safeBinaryRegression* (version: 0.1-3), *MASS* (version 7.3) and in-house-scripting. *Ggplot2* (version: 3.2.1) was used to illustrate forest plots. In multivariate logistic regression analyses, all parameters which are shown in the respective figure were considered for the estimation of the Odds ratios (OR).

Logistic regression of ROC curves and AUC analysis was calculated by the use of *pROC* (version 1.16.2). The significance level of differences in AUC values were calculated via the DeLong’s test. Survival analysis was performed with the *survival* package (version: 3.1-8). The time-to-event was defined as the difference between the date of cardiac catherization or date of the initial cancer diagnosis to the date of death or survival by October 1, 2020, respectively. The log-rank test was used to determine differences in survival. A *p*-value < 0.05 was considered significant. Cox proportional hazard ratios (HR) with 95% confidence intervals were calculated within the *survival* package (version 3.1-8) and *survminer* package (version 0.4.9).

## Results

40,329 patients underwent cardiac catherization from January 2006 to December 2017 at University Hospital Heidelberg. 6044 patients were diagnosed with a malignant disease. In 4340 patients, the initial cancer diagnosis was prior to cardiac catherization. 3666 cancer patients with available data for outcome were included in the study. Patients who were diagnosed with cancer after cardiac catherization were excluded from the study.

**Fig. 1:**
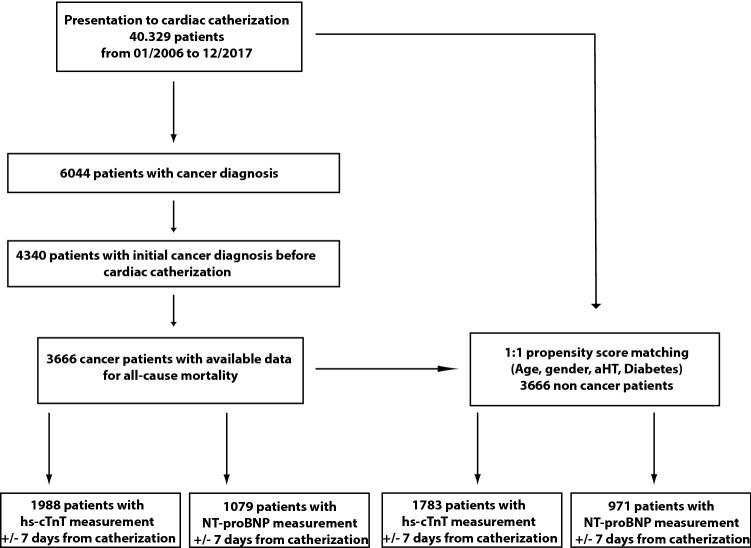
Study design. Flowchart to demonstrate the selection of the study population. 40,329 patients underwent cardiac catherization from 01/2006 to 12/2017. 6044 of these patients were diagnosed with a malignant disease. In 4340 patients, the initial cancer diagnosis was prior to cardiac catherization. Outcome data were available for 3666 patients. These patients were matched (1:1 propensity score matching) to non-cancer patients from the same cohort of 40,329 patients. The numbers of patients with measurements of hs-cTnT and NT-proBNP ± 7 days from cardiac catherization are indicated. *aHT* arterial hypertension; *NCT* National tumor centre; *hs-cTnT* high-sensitivity cardiac troponin T; *NT-proBNP* N-terminal brain natriuretic peptide

We performed a 1:1 propensity score matching of non-cancer patients according to age, gender, diabetes and arterial hypertension, who were admitted to catherization during the same period of time. The total study cohort consisted of 7332 patients. The baseline characteristics were shown in Table 1. In the cancer patient cohort, hs-cTnT was measured in 1988 and NT-proBNP in 1079 patients. In non-cancer patients, 1783 hs-cTnT measurements and 971 NT-proBNP measurements were performed (Fig. 1). In 909 cancer patients and in 741 non-cancer patients, NT-proBNP and hs-cTnT values were measured together in the given timeframe.

**Table 1:** Baseline characteristics of the study cohort

	Total patients (*n* = 7332)	Non-cancer patients (*n* = 3666)	Cancer patients (*n* = 3666)	*p*-value
Age [years] (mean± SD)	78.7 ± 10.2	78.7 ± 10.2	78.7 ± 10.3	0.883
Men (%)	4700 (64.1%)	2350 (64.1%)	2350 (64.1%)	1.00
Medical history				
Arterial hypertension	5342 (72.9%)	2674 (72.9%)	2668 (72.8%)	0.896
Diabetes	2042 (27.9%)	1020 (27.8%)	1022 (27.9%)	0.979
Skin tumor/melanoma	683 (9.3%)	0	683 (18.6%)	< 0.001
Breast cancer	460 (6.3%)	0	460 (12.6%)	< 0.001
Prostate cancer	446 (6.1%)	0	446 (12.2%)	< 0.001
GI tumor	269 (3.7%)	0	269 (7.4%)	< 0.001
Lymphoma	198 (2.7%)	0	198 (5.4%)	< 0.001
Kidney tumor	147 (2.0%)	0	147 (4.0%)	< 0.001
Neural tumor	130 (1.8%)	0	130 (3.6%)	< 0.001
Lung carcinoma	121 (1.7%)	0	121 (3.4%)	< 0.001
Leukemia	89 (1.2%)	0	89 (2.4%)	< 0.001
Blatter cancer	84 (1.1%)	0	84 (2.2%)	< 0.001
Plasmocytoma	78 (1.1%)	0	78 (2.2%)	< 0.001
Other tumor	961 (13.1%)	0	961 (26.2%)	< 0.001
Oncologic therapy				
Single chemotherapy	343 (4.7%)	0	343 (9.4%)	< 0.001
Radiotherapy	1009 (13.8%)	0	1009 (27.6%)	< 0.001
Operation	1042 (14.2%)	0	1042 (28.4%)	< 0.001
Stemcell transplantation	44 (0.6%)	0	44 (1.2%)	< 0.001
LVEF				
pEF	2868 (39.1%)	1462 (39.9%)	1406 (38.4%)	0.188
mrEF	1747 (23.8%)	895 (24.4%)	852 (23.2%)	0.250
rEF	2334 (31.8%)	1104 (30.1%)	1230 (33.6%)	0.001
Proximal coronary stenoses (≥ 90%)				
High-grade stenosis (LCA)	161	89	72	0.203
High-grade stenosis (LCX)	237	134	103	0.048
High-grade stenosis (RCA)	678	362	316	0.069
PCI	2174 (29.7%)	1141 (31.1%)	1033 (28.2%)	0.006
ACS	497 (6.8%)	280 (7.6%)	217 (5.9%)	0.004
Lab. results				
Creatinine [mg/dL] (median, IQR)	0.9 (0.72; 1.0)	1.0 (1.0; 1.0)	0.83 (0.7; 0.96)	< 0.001
Hb [mg/dL] (median, IQR)	13.0 (11.5; 14.2)	13.4 (12.1; 14.5)	12.6 (11.0; 13.9)	< 0.001
Leukocytes [/nL] (median, IQR)	8.14 (6.52; 10.45)	8.3 (6.75; 10.56)	7.9 (6.32; 10.31)	< 0.001
CRP [mg/dL] (median, IQR)	13 (5; 39)	12 (4; 32)	15 (6; 42)	0.082
Hs-cTnT [ng/L] (median, IQR)	63 (20; 348)	59 (19; 409)	66 (22; 309)	0.520
NT-proBNP [ng/L] (median, IQR)	779 (209; 3357)	668 (179; 2704)	881 (254; 3983)	< 0.001
All-cause mortality	3440 (46.9%)	1442 (39.3%)	1998 (54.5%)	< 0.001
Median survival [years] (IQR)	79.12 (72.45; 84.46)	79.13 (72.88; 84.72)	79.09 (72.03; 84.25)	0.172
Median survival after angiography [years] (IQR)	2.67 (0.75; 5.56)	2.67 (0.75; 5.58)	2.67 (0.73; 5.54)	0.633
Median survival after cancer diagnosis [years] (IQR)	8.39 (3.96; 13.89)	–	8.39 (3.96; 13.89)	–
Median time difference from cancer diagnosis to catherization [years] (IQR)	5.13 (1.5; 10.61)	–	5.13 (1.5; 10.61)	–

*ACS* acute coronay syndrome, *CRP* C-reactive protein, *GI tumor* gastrointestinal tumor, *Hb* hemoglobin, *Hs-cTnT* high-sensitivity cardiac troponin T, *NT-proBNP* N-terminal brain natriuretic peptide

The median age of the study cohort was 78.7 years, 64.1% of the patients were male, 72.9% were diagnosed with arterial hypertension and 27.9% were diabetic. 18.6% of the oncological patients were diagnosed with skin tumors or melanoma, 12.6% with breast cancer, 12.2% with prostate cancer, 7.4% with gastrointestinal (GI) tumors and 5.4% with lymphomas. For cancer patients, we found slightly lower levels of hemoglobin (Cancer patients median: 12.6 mg/dL (IQR: 11.0; 13.9 mg/dL); Non-cancer patients median: 13.4 mg/dL (IQR: 12.1; 14.5 mg/dL), *p* < 0.001) and leucocytes (Cancer patients median: 7.9/nL (IQR: 6.32; 10.3/nL); Non-cancer patients median: 8.3/nL (IQR: 6.75; 10.56/nL), *p* < 0.001). The levels of C-reactive protein (CRP) did not differ significantly between both groups (Cancer patients median: 15 mg/dL (IQR: 4; 32 mg/dL); Non-cancer median: 12 mg/dL (IQR: 6; 42 mg/dL), *p* = 0.082) (see also Table 1).

### Reduced left ventricular ejection fraction in cancer- and non-cancer patients

2868 non-cancer patients (39.1%) had a preserved LVEF, 23.8% a mildly reduced LVEF (mrEF) and 2334 patients (31.8%) a reduced LVEF (rEF). In the oncological patient cohort, we found a comparable degree of 38.4% of patients with a preserved LVEF and 23.2% of patients with mrEF. The proportion of patients with rEF was slightly higher in cancer patients (33.6%) (*p* = 0.001, chi-squared test) (Table 1). A reduced LVEF was associated with a poor outcome for both, non-cancer and cancer patients, whereas the 5-year mortality of cancer patients was generally higher (48.9% vs 68.0%, *p* < 0.001, log-rank test). In the five most prevalent entities, we saw a comparable effect of the reduced cardiac function on ACM (5-year mortality rate: melanoma/skin tumor, pEF: 49.2%, rEF: 65.6%; breast cancer, pEF: 44.2%, rEF: 60.0%; prostate cancer, pEF: 45.6%, rEF: 65.6%; GI tumor, pEF: 52.7%, rEF: 75.9%; lymphoma: pEF: 46.7%, rEF: 62.8%, *p* < 0.001, log-rank test) (Supplemental Fig. 2).

### Coronary artery disease in cancer patients

**Fig. 2:**
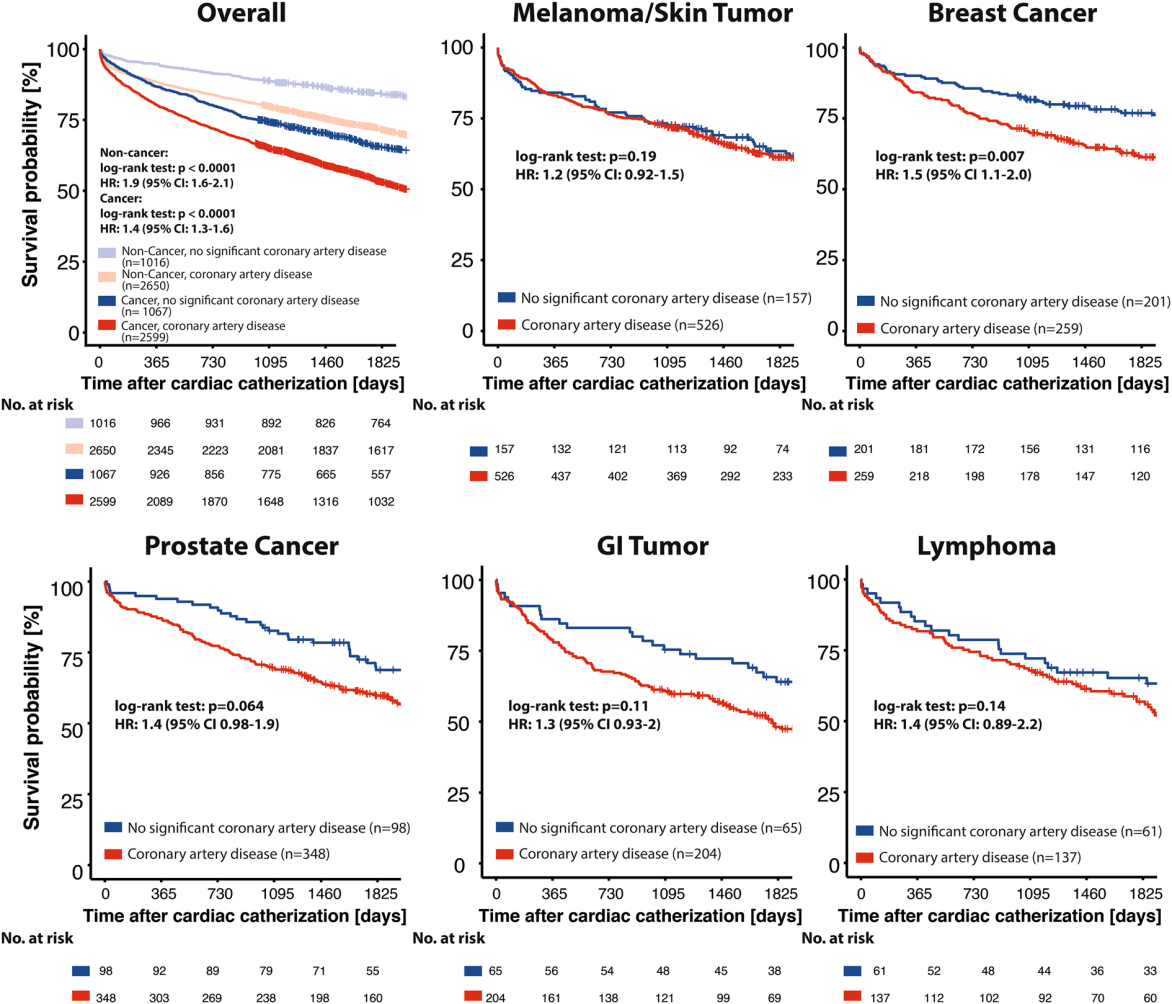
Coronary artery disease and all-cause mortality. Kaplan-Meier survival analysis for 5-year survival after cardiac catherization. Patients were grouped according to the occurrence of coronary artery disease (CAD) (≥ 50% stenosis in any segment). The overall cohort is shown at the upper left (Cancer patients in dark color, *n* = 3666; Non-cancer patients in light color, *n* = 3666). Subgroups are selected according to the five most prevalent tumor entities in our cohort (melanoma/skin tumors, *n* = 683; breast cancer, *n* = 460; prostate cancer, *n* = 446; GI tumors, *n* = 269; lymphomas, *n* = 198). Log-rank test *p*-value and hazard ratio (HR) with 95% confidence interval (CI) as indicated. *GI tumor* gastrointestinal tumor

2599 cancer patients in our cohort (70.9%) were diagnosed with CAD and no significant stenosis was found in 1067 patients (29.1%). In the non-cancer control group, the proportion of CAD was slightly higher, namely 2650 patients (72.3%). The 5-year survival of patients with CAD was significantly reduced in comparison to patients without relevant lesions both in cancer- and non-cancer patients (Cancer, no-CAD: 47.8%, CAD: 60.3%; Non-cancer, no-CAD: 24.8%, CAD: 39.0%; *p* < 0.0001, log-rank test), whereas cancer patients had higher mortality rates in general (Fig. 2).

Focusing on the five most prevalent entities in our cohort (melanoma/skin tumor, *n* = 683; breast cancer, *n* = 460; prostate cancer, *n* = 446; GI tumor, *n* = 269 and lymphoma, *n* = 198), there was a correlation of CAD and ACM in prostate cancer (no-CAD: *n* = 98; 5-year ACM: 43.9%, CAD: *n* = 348; 5-year ACM: 54.0%, *p* = 0.064, log-rank test), GI tumors (no-CAD: *n* = 65; 5-year ACM: 41.5%, CAD: *n* = 204; 5-year ACM: 66.2%, *p* = 0.11, log-rank test), and lymphomas (no-CAD: *n* = 61; 5-year ACM: 45.9%, CAD: *n* = 137; 5-year ACM: 56.2%, *p* = 0.14, log-rank test) without reaching statistical significance. In patients with melanoma/skin tumors (no-CAD: *n* = 157; 5-year ACM: 52.9%, CAD: *n* = 526; 5-year ACM: 55.7%, *p* = 0.19, log-rank test), there was no correlation between CAD and ACM. In contrast, patients with breast cancer and CAD had a significantly higher ACM than breast cancer patients without CAD (no-CAD: *n* = 201; 5-year ACM: 42.3%, CAD: *n* = 259; 5-year ACM: 53.7%, *p* = 0.007, log-rank test) (Fig. 2).

**Fig. 3:**
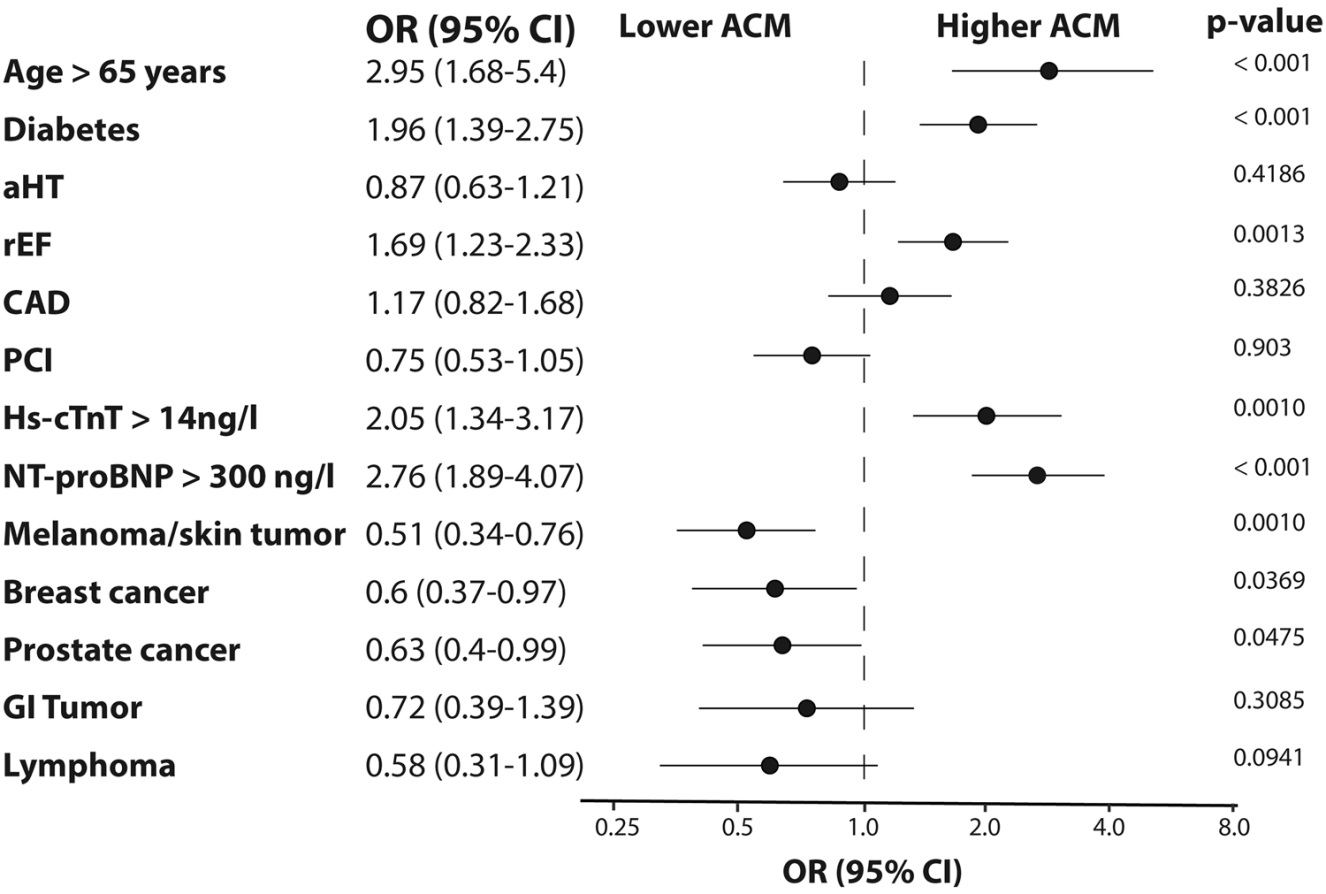
Determinants of all-cause mortality. Forest plot of multivariate logistic regression analysis for all-cause mortality (ACM). Odds ratio (OR) with 95% confidence interval (CI) and *p*-value as indicated. *aHT* arterial hypertension, *CAD* coronary artery disease, *GI tumor* gastrointestinal tumor, *rEF* reduced ejection fraction, *hs-cTnT* high-sensitivity cardiac troponin T, *NT-proBNP* N-terminal brain natriuretic peptide, *PCI* percutaneous coronary intervention

In multivariate logistic regression analysis, CAD and percutaneous coronary intervention (PCI) were no independent parameters to predict ACM (CAD: *p* = 0.38; PCI: p = 0.9). However, elevated hs-cTnT (OR: 2.05, *p* = 0.001) or NT-proBNP (OR: 2.76, *p* < 0.001) or a reduced LVEF (OR: 1.69, *p* = 0.0013) was associated independently with mortality. Patients with breast cancer, skin tumors and prostate cancer showed a more favorable outcome. Age > 65 years and diabetes correlated with a poor outcome (Fig. 3).

### Elevation of cardiac biomarkers in cancer patients

We further evaluated hs-cTnT and NT-proBNP values between 7 days before and 7 days after cardiac catherization. Overall, cancer and non-cancer patients did not differ significantly in maximal hs-cTnT values (*p* = 0.52, Wilcoxon-rank sum test). NT-proBNP was found to be significantly higher in cancer patients (881 ng/L in cancer patients, 668 ng/L in non-cancer patients, *p* < 0.001, Wilcoxon-rank sum test). Elevated levels of hs-cTnT (above 14 ng/L) and of NT-proBNP (above 300 ng/L) were associated with higher rates of 5-year ACM. This was the case for cancer patients (hs-cTnT > 14 ng/L: 64.6%, *p* < 0.0001, log-rank test; NT-proBNP > 300 ng/L: 62.9%, *p* < 0.0001, log-rank test) and non-cancer patients (hs-cTnT > 14 ng/L: 50.0%, *p* < 0.0001, log-rank test; NT-proBNP > 300 ng/L: 44.2%, *p* < 0.0001, log-rank test). Among cancer patients, skin tumor (hs-cTnT ≤ 14 ng/L: *n* = 63; 5-year ACM: 42.9%, hs-cTnT > 14 ng/L: *n* = 328; 5-year ACM: 61.9%, log-rank test: *p* < 0.0001), breast cancer (hs-cTnT ≤ 14 ng/L: *n* = 50; 5-year ACM: 54.0%, hs-cTnT > 14 ng/L: *n* = 170; 5-year ACM: 59.4%, log-rank test: *p* = 0.0003), prostate cancer (hs-cTnT ≤ 14 ng/L: *n* = 34; 5-year ACM: 23.5%, hs-cTnT > 14 ng/L: *n* = 217; 5-year ACM: 60.8%, log-rank test: *p* = 0.0003) and lymphomas (hs-cTnT ≤ 14 ng/L: *n* = 13; 5-year ACM: 23.1%, hs-cTnT > 14 ng/L: *n* = 93; 5-year ACM: 63.1%, log-rank test: *p* = 0.045), patients with hs-cTnT > 14 ng/L had a significantly higher 5-year ACM (Supplemental Fig. 3). Elevated values of NT-proBNP (> 300 ng/L) could be linked with a poor outcome in patients with skin tumor (NT-proBNP ≤ 300 ng/L: *n* = 70; 5-year ACM: 44.3%, NT-proBNP > 300 ng/L: *n* = 139; 5-year ACM: 61.2%, log-rank test: *p* < 0.0001), breast cancer (NT-proBNP ≤ 300 ng/L: *n* = 46; 5-year ACM: 37%, NT-proBNP > 300 ng/L: *n* = 100; 5-year ACM: 46.0%, log-rank test: *p* = 0.00016), prostate cancer (NT-proBNP ≤ 300 ng/L: *n* = 40; 5-year ACM: 27.5%, NT-proBNP > 300 ng/L: *n* = 102; 5-year ACM: 56.9%, log-rank test: *p* = 0.00035), GI tumors (NT-proBNP ≤ 300 ng/L: *n* = 26; 5-year ACM: 42.3%, NT-proBNP > 300 ng/L: *n* = 48; 5-year ACM: 66.7%, log-rank test: *p* = 0.026) and lymphomas (NT-proBNP ≤ 300 ng/L: *n* = 15; 5-year ACM: 33.3%, NT-proBNP > 300 ng/L: *n* = 50; 5-year ACM: 58.0%, log-rank test: *p* = 0.028) (Supplemental Fig. 4).

### Determination of all-cause mortality in cancer patients

Considering the 5-year survival after the timepoint of catherization, all parameters—including CAD, LVEF, hs-cTnT and NT-proBNP—were able to distinguish between patients with high and low survival rates in the total cancer as well as in the non-cancer cohort. Cancer patients with non-elevated hs-cTnT (5-year ACM: 44.6%) or NT-proBNP (5-year ACM: 41.4%) showed lower mortality rates than patients with preserved LVEF (5-year ACM: 50.5%) or without a relevant CAD (5-year ACM: 47.8%). This pattern was comparable in the total cohort and in the subgroups accordingly (Fig. 2, Supplemental Figs. 2–4). In survival analysis that considered the time after the diagnosis of the malignant disease, again we found a similar discriminative role of the single factors. Especially non-elevated hs-cTnT or NT-proBNP were able to identify patients with a low mortality risk (5-year ACM: hs-cTnT 18.4% vs 24.1%, *p* < 0.0001, log-rank test; NT-proBNP 8.2% vs 15.5%, *p* < 0.0001, log-rank test) (Supplemental Fig. 5).

**Fig. 4:**
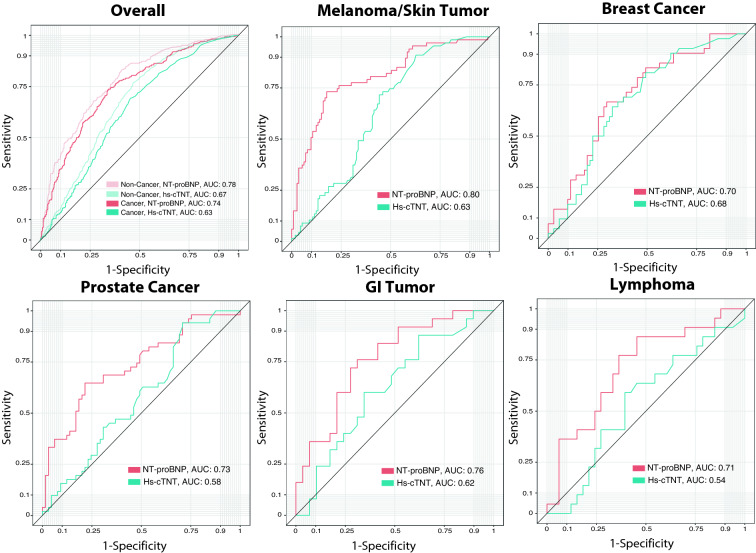
Cardiac biomarkers determine all-cause mortality. Receiver operating characteristic (ROC) curves for all-cause mortality (ACM). Patients are grouped according to hs-cTnT and NT-proBNP values. At the upper left, the overall cancer and non-cancer cohort is shown (Cancer patients in dark color; Non-cancer patients in bright color). Patients are subgrouped according to the five most prevalent malignant diseases in our cohort. Area under the curve (AUC) as indicated. *GI tumor* gastrointestinal tumor, *Hs-cTnT* high-sensitivity cardiac troponin T, *NT-proBNP* N-terminal brain natriuretic peptide

We further evaluated the predictive value of hs-cTnT and NT-proBNP for ACM using Receiver Operating Characteristic (ROC) curves. Cancer and non-cancer patients’ mortality was associated with elevated hs-cTnT and NT-proBNP. NT-proBNP, however, had a higher predictive value for ACM (Non-Cancer AUC: 0.78; Cancer AUC: 0.74) than hs-cTnT (Non-Cancer AUC: 0.67; Cancer AUC: 0.63). There were no major differences between cancer and non-cancer patients. In skin tumor patients, the predictive value of NT-proBNP was slightly better (AUC: 0.8). In breast cancer patients, we observed the lowest association of NT-proBNP and ACM (AUC: 0.70). In all of the top five entities as well as in non-cancer patients, NT-proBNP had a higher predictive value than hs-cTnT (Fig. 4). Comparing the primary indications for cardiac catherization, we observed a slightly higher proportion of non-cancer patients with CAD (*p* < 0.001, chi-squared test) and a slightly higher proportion of cancer patients with cardiomyopathy (*p* < 0.001, chi-squared test) and a pre-surgery cardiological assessment (*p* < 0.001, chi-squared test). The indications of valvular heart disease and resuscitation did not differ significantly (Supplemental Fig. 6).

## Discussion

### Is the cardiovascular morbidity and mortality of cancer patients underestimated?

In the present study, we evaluated the impact of cardiac comorbidity on ACM in cancer patients who underwent invasive diagnostic procedures, considering LVEF, CAD and biomarkers. Both, cancer and cardiovascular diseases, are a major burden in the western world. [21] Not solely oncological therapies, but cancer itself has a negative impact on cardiovascular diseases and vice versa. [22, 23] Therefore, cancer and cardiovascular patients should not be considered separately. In the present study, cancer was diagnosed in approximately 15% (6044/40,329) of all admitted patients for cardiac catherization, most of whom were suspected of having CAD.

In the daily clinical practice, signs and symptoms of acute coronary syndrome (ACS) and heart failure (HF) are frequently seen in cancer and non-cancer patients. Nevertheless, the underlying pathologies and pathomechanism may vary due to the systemic disease itself or its therapy. Despite this prominent co-occurrence of cancer and CVD, still little is known about specific cardiac parameters for surveillance in cancer patients. In our study, the predictive value was evident at the timepoint of admission to catheterization. More importantly, we found that 5-year ACM from timepoint of the cancer diagnosis was reduced. This additionally highlights the predictive value of cardiac assessment in oncological patients.

### The comorbidity of coronary artery disease in cancer patients

CAD is highly prevalent in cancer patients, especially in patients with hematologic malignancies or lung cancer [24] or after chest radiation in breast cancer patients. [25] Unfortunately, many of them are underdiagnosed and therefore do not receive the appropriate medical therapy. [24, 26] In a large observational study in the US, 6.5 million patients with acute myocardial infarction (MI) are analyzed. Approximately 10% of these patients are diagnosed with cancer. Depending on the cancer entity and the status of metastasis, cancer patients have a worse clinical outcome compared to non-cancer patients [3]. Nevertheless, medical as well as interventional treatment strategies of MI are effective in cancer patients and improve the ACM [27]. Despite the higher risk of thromboembolism [28] or bleeding [29], cancer patients do not have a worse outcome after coronary artery bypass than a comparable non-cancer patient [30]. The periprocedural risk and outcome after PCI was also comparable. This is shown in a retrospective analysis of 153 cancer patients and 153 matched non-cancer patients. Given a comparable distribution of coronary lesions, PCI is less frequently performed in cancer patients [31].

The prevalence of cancer of 15% as well as the dominance of breast cancer, prostate cancer, melanoma, GI tumors and lymphoma—the main entities in our cohort—is in the same range as the published data of large cohorts [3, 24]. In line with the published data, we observe a lower rate of PCI in the present cancer cohort. Despite this difference in the treatment of CAD in cancer and non-cancer patients, we observe no major differences in the frequency or severity of coronary lesions between both groups. One might speculate that cancer patients in general, apart from specific entities or treatment regimens, do not have a higher risk of CAD but of CVD of other origin instead, documented by elevated cardiac biomarker and/or reduced LVEF. In our cohort of patients with a matched risk profile and no significant differences in coronary stenosis, cancer patients suffered more frequently from rEF and had higher median NT-proBNP levels. This might be explained by the fact that the proportion of cancer patients with the primary indication of suspected cardiomyopathy is slightly higher.

Cancer patients’ specific cardiovascular comorbidity may subsequently be of non-ischemic origin. A smaller number of patients with an acute coronary syndrome and individualized considerations of the risk and benefit of an intervention in a patient with malignant disease may be reasons for the lower rates of PCI.

### The role of cardiac biomarkers in cancer patients

Regarding surveillance strategies of cancer patients during systemic therapy, the role of imaging and evaluation of the LVEF is highlighted by the European Society of Cardiology (ESC) [15] and the American Society of Clinical Oncology (ASCO) [32]. Recently, explicit guidelines for the use of biomarkers in the risk stratification of cancer patients have been added [33]. In the present cohort, we clearly see the influence of impairments in LVEF on ACM, but NT-proBNP as well as hs-cTnT show an independent association on ACM in multivariate logistic regression. The association of biomarkers and ACM is found to be even more pronounced than the association of LVEF and ACM which underpins the potential role of biomarkers for surveillance and risk stratification. In recent years, some studies focused on the role of cardiac biomarkers in different cancer patient cohorts [34–42]. To our knowledge, we provide the so far largest dataset that compares the influence of multiple factors side by side, including LVEF, biomarkers and CAD.

## Conclusions

Cardiac surveillance of cancer patients mostly focuses on the detection of echocardiographic impairments in LVEF during chemotherapy [8, 43]. Apart from well-known cardiotoxic effects, there are more recent studies highlighting the possible value of cardiac biomarkers to detect cardiovascular comorbidity in cancer patients.

In the present study, we evaluate the LVEF, the coronary artery stenoses and cardiac biomarkers (hs-cTnT and NT-proBNP) in a large representative cohort of cancer patients. Reduced LVEF serves as an important prognostic parameter, whereas the occurrence of CAD correlates with the ACM especially in breast cancer patients. In addition, higher levels of NT-proBNP as a general marker for heart failure and/or cardiac stress response are associated with higher ACM.

Given these data, hs-cTnT and NT-proBNP should be considered to be part of surveillance strategies of cancer patients.

### Study limitations

There are several potential limitations to the present study. This is a monocentric, retrospective case control study, which may have led to a selection bias. Cardiac catherizations of the study cohort were performed over a period of eleven years. During this time, there may have been slight differences in the catherization procedure, even though standardized methods were used at the institution. We evaluated the influence of the cardiac risk factors diabetes and arterial hypertension on ACM and performed propensity-score matching between cancer and non-cancer patients according to age, gender and these two factors. There was a lack of data concerning other cardiac risk factors, such as smoking and lipid profiles. Further, there was lack of data concerning the occurrence of arrhythmic events, such as atrial fibrillation, in the patient cohort. We had access to reliable data of ACM but no data on cause of death, e.g., due to cardiovascular or oncological causes.

## Supplementary Information

Below is the link to the electronic supplementary material.

Supplementary file1 (PDF 4178 KB)
