# Supplementary material for: Coronary artery disease, left ventricular function and cardiac biomarkers determine all-cause mortality in cancer patients—a large monocenter cohort study
Source: Clin Res Cardiol. 2022 Mar 21;112(2):203–14. doi: 10.1007/s00392-022-02001-6 (PMC9898338; doi:10.1007/s00392-022-02001-6)
Supplement: Supplementary file 1 — Supplementary file1 (PDF 4178 KB) [file 392_2022_2001_MOESM1_ESM.pdf]

Supplemental Data

Coronary artery disease, left ventricular function and cardiac biomarkers determine all-cause mortality in cancer patients - a large monocenter cohort study

Daniel Finke[1,2], Markus B. Heckmann[1,2], Susanna Wilhelm[1], Lukas Entenmann[1], Hauke Hund[1], Nina Bougatf [3], Hugo A. Katus[1,2], Norbert Frey[1,2], Lorenz H. Lehmann[1,2,4*]

[1] Department of Cardiology, Heidelberg University Hospital, Heidelberg, Germany
[2] German Centre for Cardiovascular Research (DZHK), partner site Heidelberg/Mannheim, Germany
[3] Nationales Tumorzentrum, Heidelberg (NCT)
[4] Deutsches Krebsforschungszentrum, Heidelberg (DKFZ)

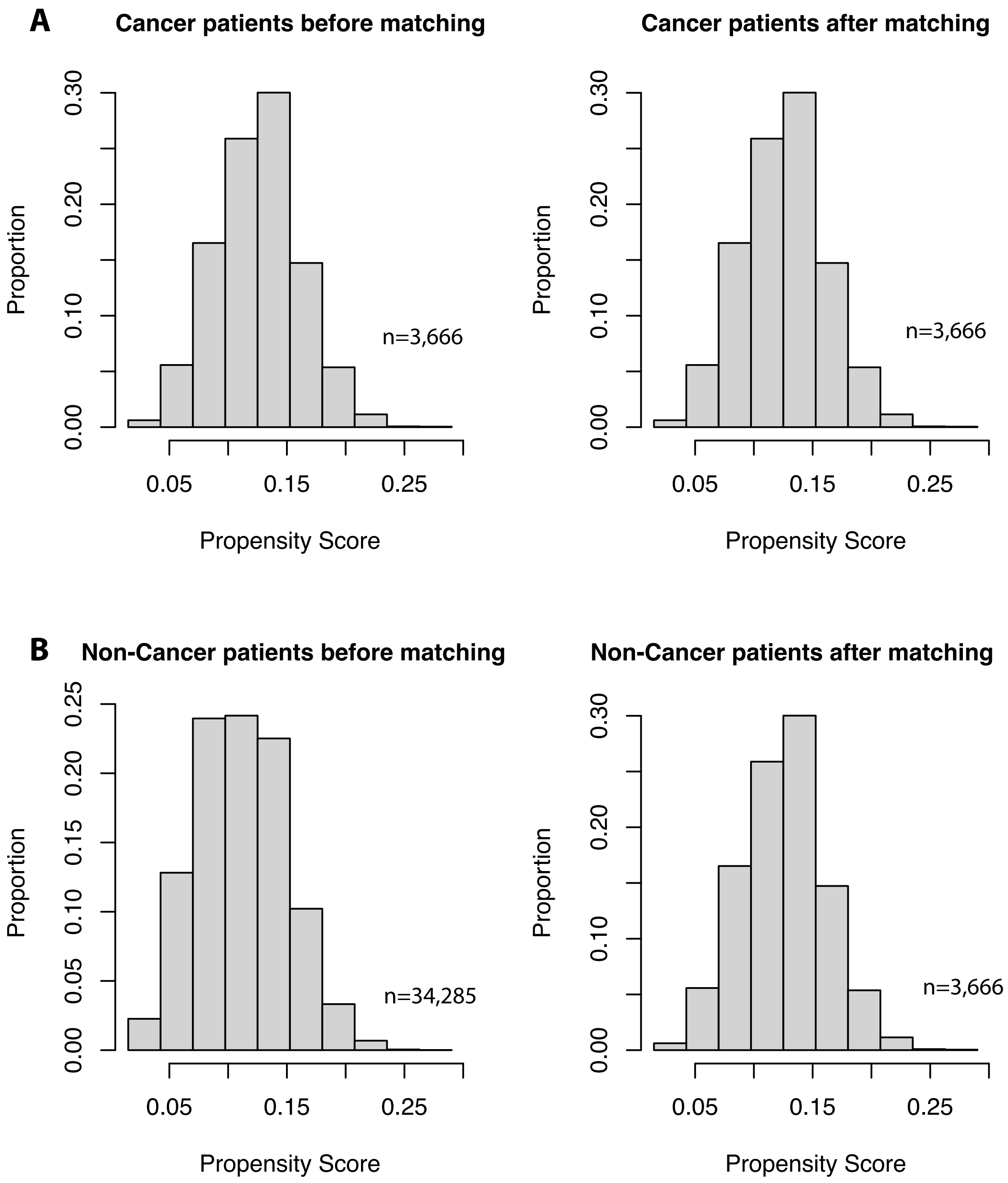

Supplemental Figure 1:
Propensity score matching of Non-Cancer patients to Cancer-patients. Distribution of propensity scores before and after matching in (A) Cancer and (B) Non-Cancer patients. Number of patients and scores as indicated.

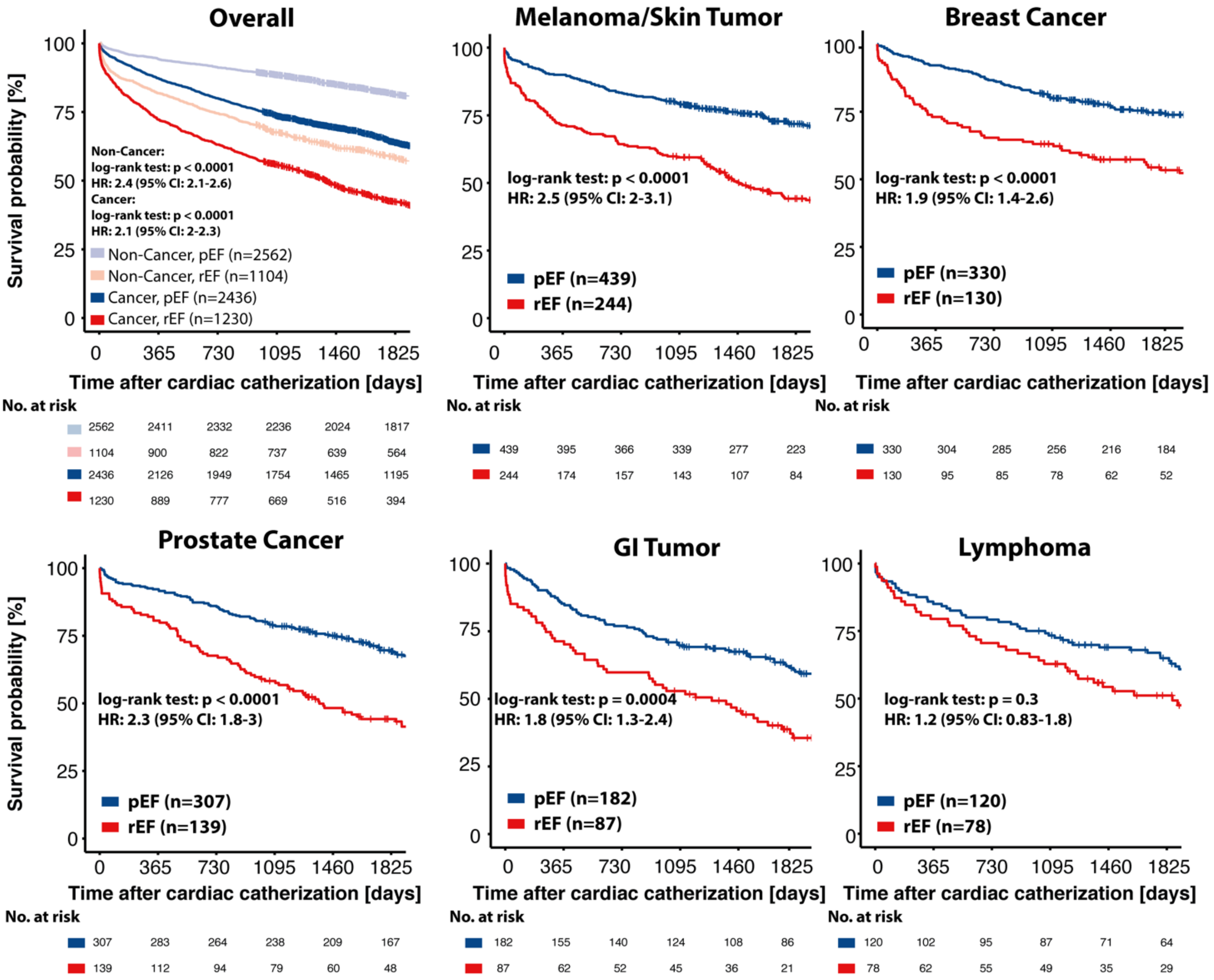

Supplemental Figure 2:

Kaplan-Meier survival analysis for 5-year-survival after cardiac catherization. Patients are grouped according to preserved (pEF) and reduced (rEF) systolic function. The overall cohort is shown at the upper left (Cancer patients in dark color, n= 3,666, Non-cancer patients in light color, n= 3,666). Subgroups are selected according to the five most prevalent tumor entities in our cohort (melanoma/skin tumors, n=683; breast cancer, n=460; prostate cancer, n=446, GI tumor, n=269, lymphoma, n=198).

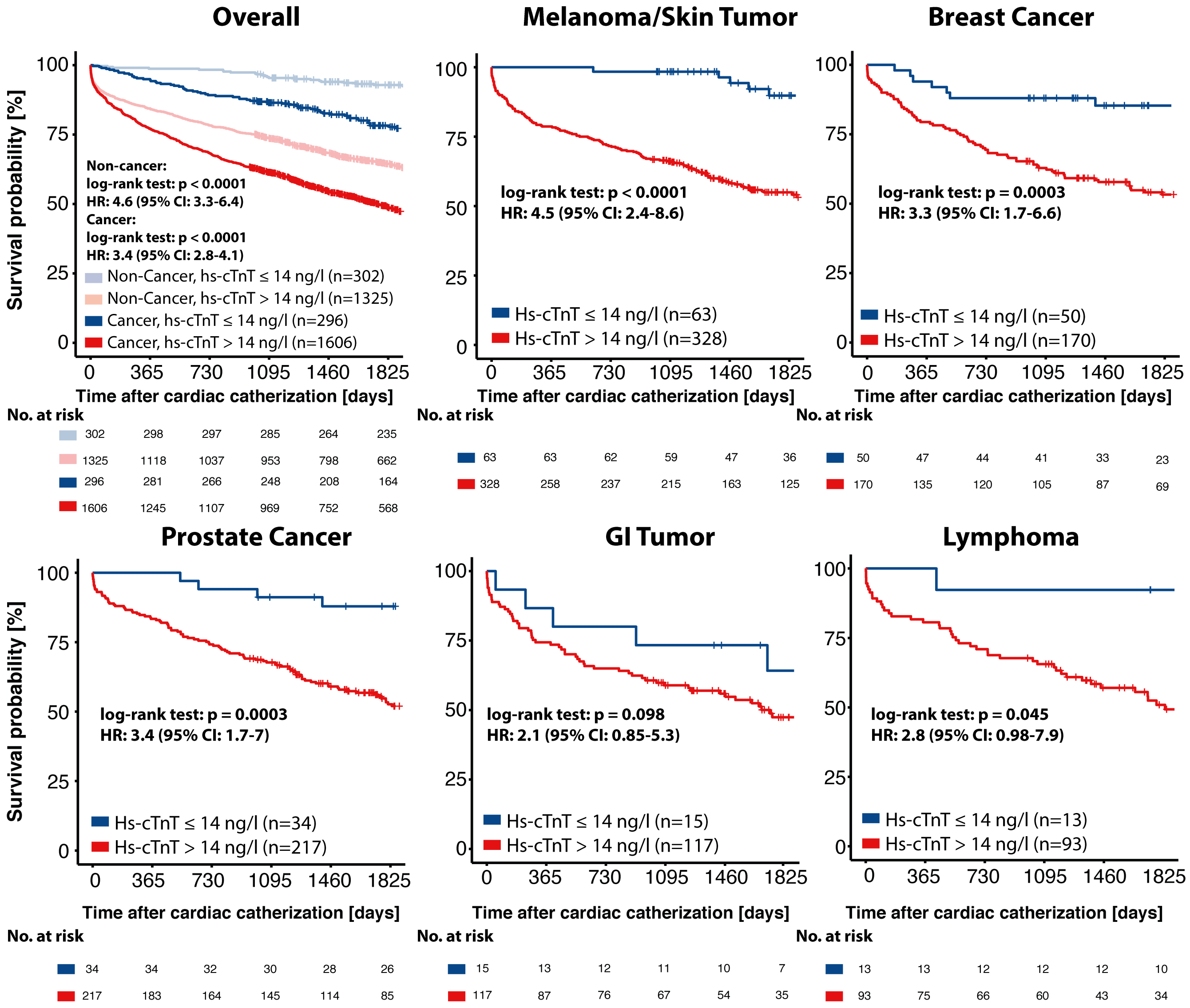

Supplemental Figure 3:
Kaplan-Meier survival analysis for 5-year-survival after cardiac catherization. Patients are grouped according to elevated hs-cTnT (> 14 ng/l) and non-elevated hs-cTnT (≤ 14 ng/l). The overall cohort is shown at the upper left (Cancer patients in dark color, n= 3666, Non-cancer patients in light color, n= 3666). Subgroups are selected according to the five most prevalent tumor entities in our cohort (melanoma/skin tumors, n=683; breast cancer, n=460; prostate cancer, n=446, GI tumor, n=269, lymphoma, n=198).

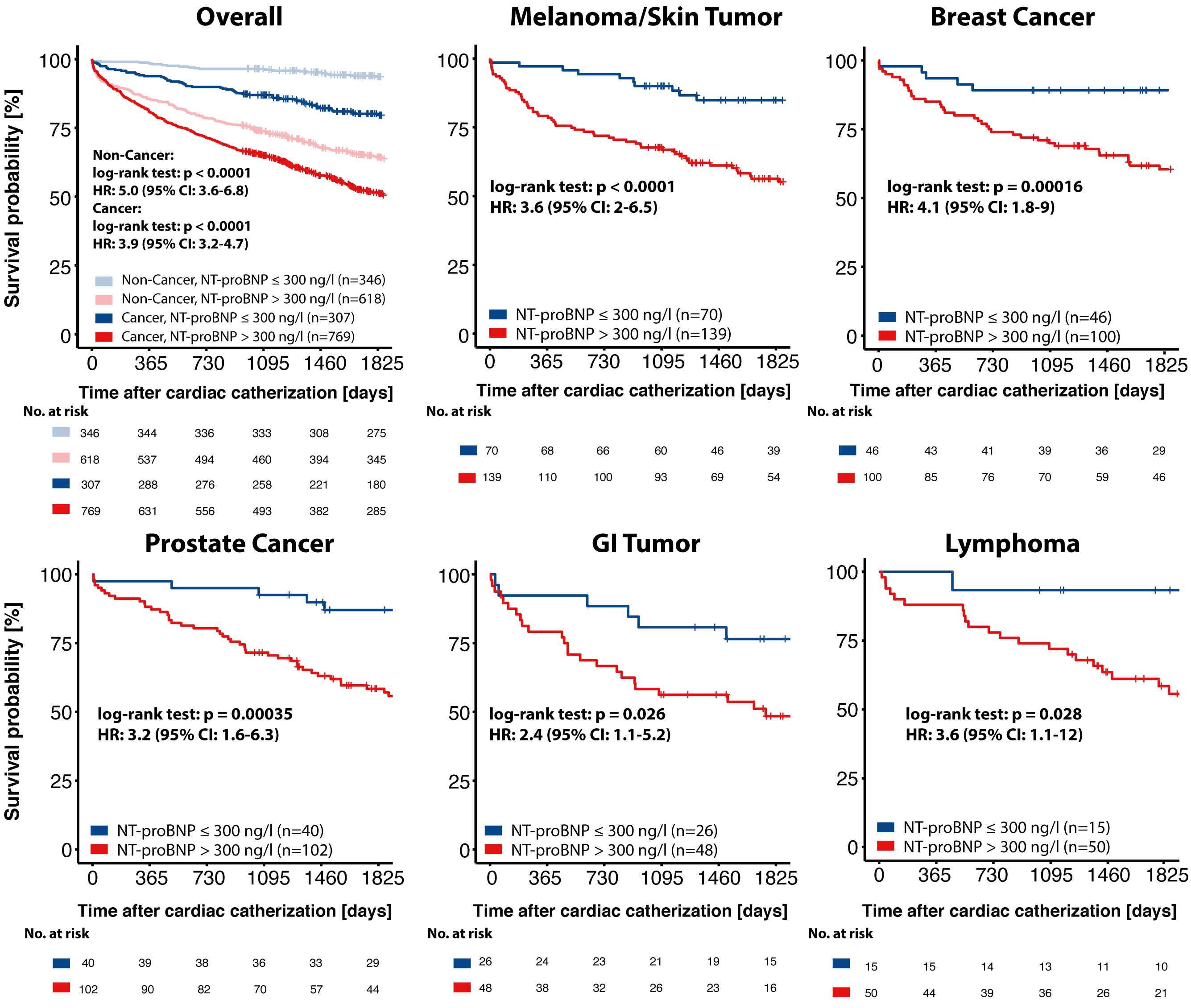

Supplemental Figure 4:
Kaplan-Meier survival analysis for 5-year-survival after cardiac catherization. Patients are grouped according to elevated NT-proBNP (> 300 ng/l) and non-elevated NT-proBNP (≤ 300 ng/l). The overall cohort is shown at the upper left (Cancer patients in dark color, n= 3666, Non-cancer patients in light color, n= 3666). Subgroups are selected according to the five most prevalent tumor entities in our cohort (melanoma/skin tumors, n=683; breast cancer, n=460; prostate cancer, n=446, GI tumor, n=269, lymphoma, n=198).

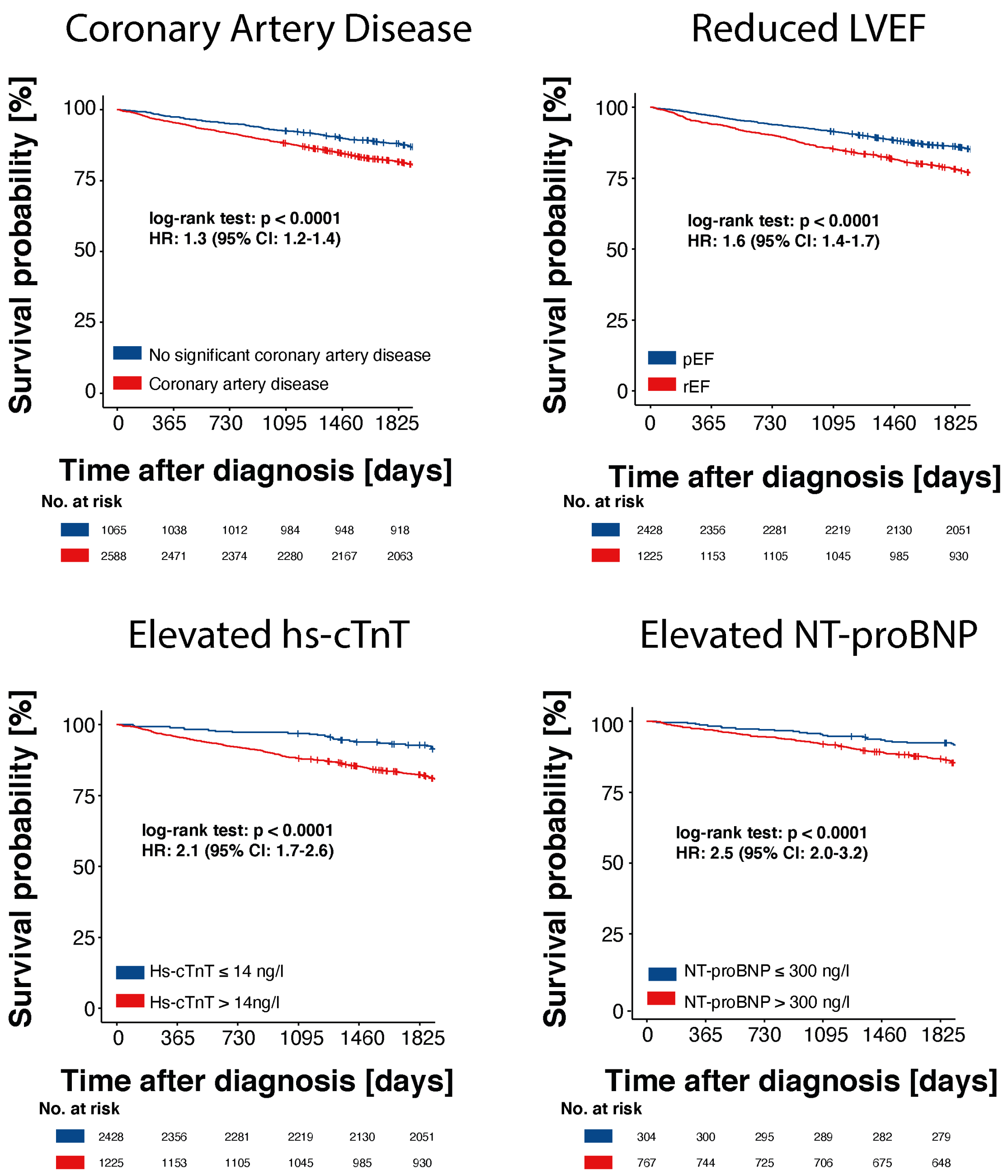

Supplemental Figure 5:
Kaplan-Meier survival analysis for 5-year-survival after the initial cancer diagnosis. Cancer patients are grouped according to the occurrence of coronary artery disease (upper left panel), reduced left ventricular ejection fraction (LVEF) (upper right panel), elevated hs-cTnT (> 14 ng/l) and non-elevated hs-cTnT (≤ 14 ng/l) (lower left panel) and elevated NT-proBNP (> 300 ng/l) and non-elevated NT-proBNP (≤ 300 ng/l) (lower right panel).

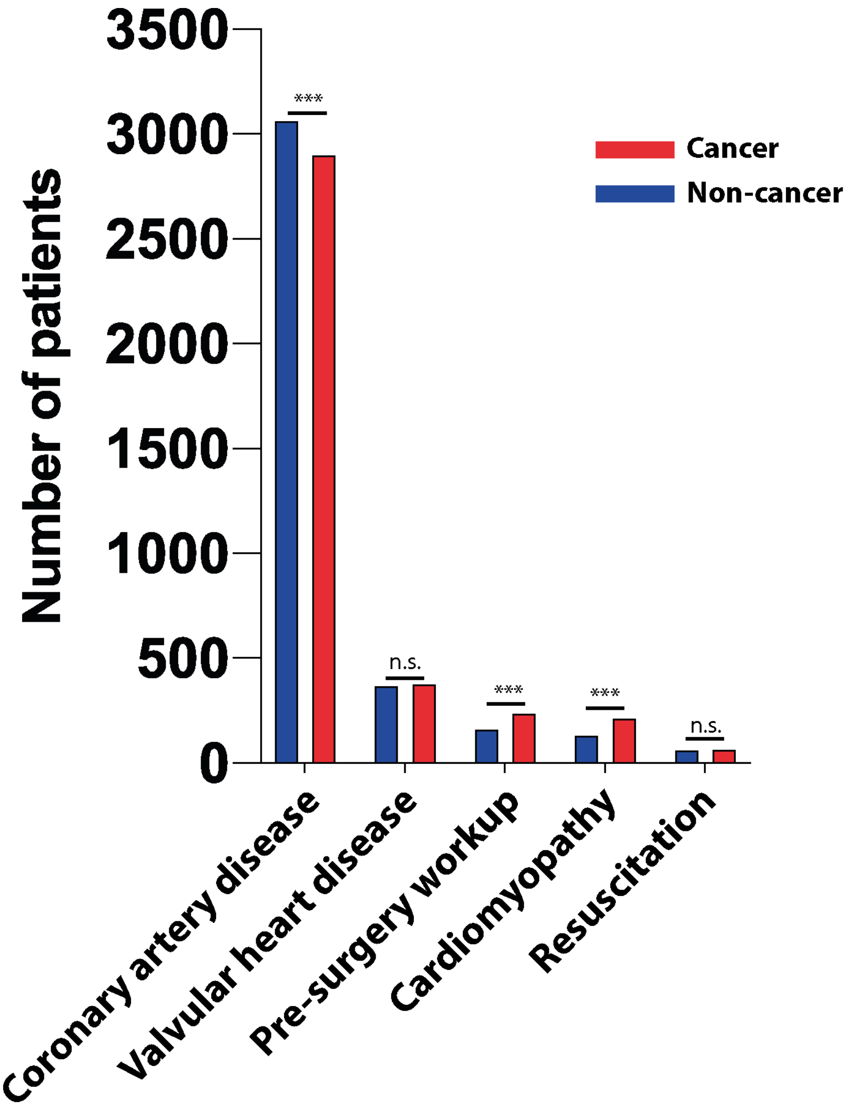

Supplemental Figure 6:
Distribution of the primary cardiac catherization indications in cancer and non-cancer patients. Number of patients that were subjected to catherization due to coronary artery disease, valvular heart disease, as a pre-surgery workup, suspected cardiomyopathy or after resuscitation as indicated. Chi-squared test: n.s.: non-significant, *: $p< 0.05$, **: $p< 0.01$, ***: $p< 0.001$.
